# Estimating radiation exposure of the brain of a physician with a protective flap in interventional radiology: A phantom study

**DOI:** 10.1002/acm2.13532

**Published:** 2022-01-19

**Authors:** Shota Hattori, Hajime Monzen, Mikoto Tamura, Hiroyuki Kosaka, Yasunori Nakamura, Yasumasa Nishimura

**Affiliations:** ^1^ Department of Medical Physics Graduate School of Medical Sciences Kindai University Osakasayama Osaka Japan; ^2^ Department of Radiological Center Kindai University Hospital Osakasayama Osaka Japan; ^3^ Department of Radiation Oncology Faculty of Medicine Kindai University Osakasayama Osaka Japan

**Keywords:** brain exposure, interventional radiology, occupational exposure, protective flap, radiation protection

## Abstract

**Purpose:**

The efficiency of protective equipment for the brain has not been verified at the left anterior oblique (LAO) position, which is commonly used in clinical procedures. The purpose of this study was to investigate radiation exposure of the brain in interventional radiology (IR) and the shielding ability of a new protective flap.

**Methods:**

We made a flap that combined a protective cap with a left lateral face shield. The flap was made of tungsten‐containing rubber (TCR). An anthropomorphic head phantom was placed at the physician's position, and air kerma rates (μGy/min and μGy/15s) were measured by electronic dosimeter at three locations: the surface of the left side of the head, and the left and right temporal lobes with the protective cap and the flap in fluoroscopy and cine modes. The X‐ray tube was at the lower left side of the physician, and its angles were LAO60 and LAO60CAU40. The tube voltage (95–125 kV), tube current (4.7–732 mA), and air kerma rate (27.8–1078 mGy/min) were automatically adjusted by the X‐ray system. We obtained the cap and the flap shielding efficiencies.

**Results:**

In cine mode at LAO60CAU40, the shielding efficiencies on the surface of the left side of the head and left temporal lobe with the cap were 92.6% and 5.1%, respectively, and the corresponding shielding efficiencies with the flap were 92.5% and 86.1%, respectively. The flap can reduce radiation exposure of the brain more than the cap alone.

**Conclusions:**

At the left anterior oblique in interventional radiology, the flap can reduce exposure to the brain.

## 1 INTRODUCTION

Interventional radiology (IR) with X‐ray equipment plays an important clinical role and is used all over the world. Despite advances in diagnostic and treatment technology, radiation exposure is a proven hazard to patients and a potential hazard for physicians and nurses in IR. The patient exposure per procedure ranges from 2.5 to 5.0 mSv for diagnostic coronary angiography.^1^ Aspect such as tube voltage, tube current, and filters are managed to reduce the patient exposure per procedure as much as possible. However, Picano E et al. reported that the annual exposure of a cardiologist under a lead apron is 5 mSv.^2^ Cardiologists are exposed to scatter radiation from the patient, so they use lead aprons and lead glasses to protect them during procedures.^2^, ^3^, ^4^, ^5^

Roguin et al. reported an increased risk of brain cancers in interventional physicians.^6^ The majority of these cancers were glioblastoma multiforme, identified in 17 of 31 cases, with two cases of astrocytoma and five of meningioma. In 26 of 31 cases, data were available regarding the side of the brain involved. The malignancy was on the left side in 22, at the midline in one, and on the right side in three physicians. Glioblastoma multiforme is mostly a central cancer, but in this report, 85% of the cancers developed on the left side. There are also reports on the risk of radiation exposure of the physician's brain.^7^, ^8^ Rajaraman et al. reported an increased risk of brain cancer among radiologists working in IR.^9^ In addition, Stahl et al. concluded that more clearly established are the risks of radiation to the fetus and the risk of cataracts in interventional cardiologists and interventional radiologists. Interventionalists can mitigate these risks by following established radiation safety practices.^10^ Although stochastic effects such as cancer induction by low radiation doses have not yet been clarified, there are reports of such risks.^11^

Furthermore, Picano et al. reported that annual exposure of a physician's head ranges from 20 to 30 mSv, which is 10–20 times higher than the exposure recorded beneath the apron.^2^ Attention is given to protecting the head from radiation exposure. Lead shields are used to reduce radiation exposure, but the head is not completely shielded.^12^, ^13^ A head‐protecting surgical cap is commercially available, and there are reports on it. The Brain Radiation Exposure and Attenuation During Invasive Cardiology Procedures (BRAIN) study reported that a cap containing barium and bismuth reduced head exposure.^14^ However, Kirkwood ML et al. reported that exposure of the surface of the head is reduced, while brain exposure is not.^15^ The risk of radiation exposure of the brain can be a problem, and it is therefore important to determine this exposure.

In past studies, exposure of the brain was usually measured at only the posterior‐anterior (PA) position. At this time, we measured at the left anterior oblique (LAO) position, which is often used during IR. The aim of this study was to determine radiation exposure of a brain using an anthropomorphic head phantom into which an electronic dosimeter could be inserted at X‐ray tube angles commonly used in IR. We created a protective flap using tungsten‐containing rubber (TCR) and evaluated its shielding efficiency and usefulness for protecting the brain.

## 2 MATERIALS AND METHODS

### 2.1 Phantom setups

**FIGURE 1:**
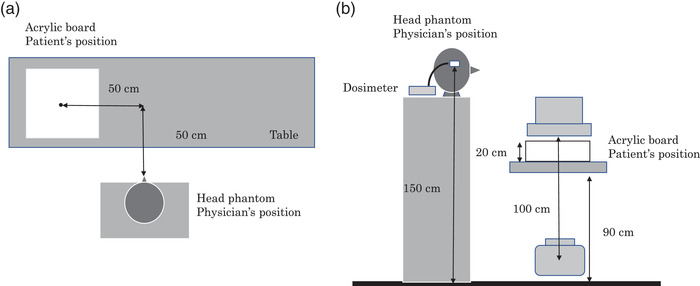
Schematic view from (a) above and (b) the side of the experimental setup

Figure 1 shows the experimental setup used to simulate the typical IR setting. In general, physicians perform their procedures standing on the right side of the patient in IR. An anthropomorphic head phantom (Alderson RANDO, Alderson Research Laboratories, Stamford, USA) was used to simulate a physician, and an acrylic board with a thickness of 20 cm was used to simulate a patient.^16^

### 2.2 X‐ray conditions

In this study, we used an X‐ray angiography system (Azurion, Philips, Netherlands) equipped with a flat‐panel detector and an under‐couch X‐ray tube. We set the source‐to‐image receptor distance to 100 cm, the height of the patient table to 90 cm, and the field of view to 20 cm. Measurements were performed in fluoroscopy and cine modes at frame rates of 7.5 f/s and 15 f/s, respectively. The tube voltage (kV), tube current (mA), and air kerma rate (AKR: mGy/min) were automatically adjusted by the X‐ray system, whose parameters are listed in Table 1. The filters for fluoroscopy and cine modes were set to 0.4 mm Cu and 1.0 mm Al. The X‐ray tube angles included left anterior oblique 60 degrees (LAO60) and left anterior oblique 60 and caudal 40 degrees (LAO60CAU40), which are typical IR conditions in our hospital.^16^, ^17^

**TABLE 1:** Parameters of the automatically configured X‐ray system

	**Fluoroscopy**			**Cine**		
**Tube angles**	**Tube voltage (kV)**	**Tube current (mA)**	**AKR (mGy/min)**	**Tube voltage (kV)**	**Tube current (mA)**	**AKR (mGy/min)**
LAO60	118	4.7	27.8	95	724–732	494–495
LAO60CAU40	120	4.7	28.8	125	561–569	1073–1078

### 2.3 Dosimetry

**FIGURE 2:**
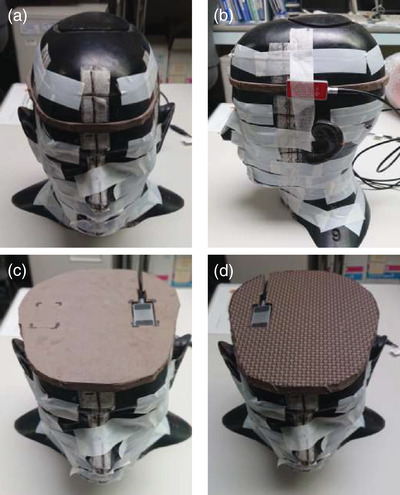
(a) Head phantom used in this study. The three measurement points on the head phantom are: (b) the surface of the left side of the head, (c) the left temporal lobe, and (d) the right temporal lobe

The AKR was measured using a diode detector (RTI Dose Probe; RTI Electronics, Mölndal, Sweden) with an electronic dosimeter (Piranha, RTI Electronics, Mölndal, Sweden).^18^ The dosimeter was calibrated with an ionization chamber (TN30013, PTW, Freiburg, Germany) according to Al filter thickness of the RQR5 (70 kV, 10 mA) radiation quality on May 3, 2021.^19^ A dosimeter was placed on the physician phantom on the surface of the left side of the head and on the centers of the left and right temporal lobes of the physician phantom (Figure 2) to measure the exposure of these areas.^7^ The surface of the left side of the head is outside the skull and is placed on the head skin. The left temporal lobe is inside the skull. The AKR (μGy/min) was measured in fluoroscopy mode. In cine mode, the AKR was measured with an acquisition time of 15 s (μGy/15s). The AKR was measured three times and the average value was calculated.

### 2.4 Characteristics of the TCR and Flap

**FIGURE 3:**
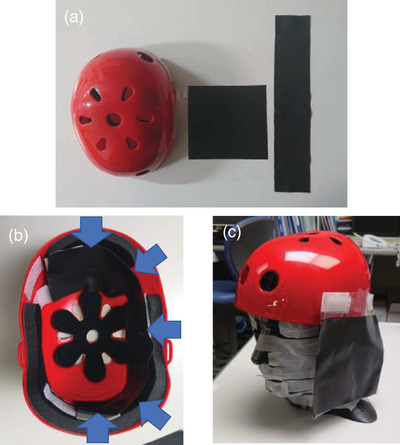
Flap using tungsten‐containing rubber (TCR). (a) Plastic helmet (left), left lateral face shield (center), and TCR used in the helmet for the cap alone (CA) (right). (b) The CA was created by affixing the TCR inside the front, left, and back of the helmet. Arrows indicate where the TCR was affixed. (c) Flap set on an anthropomorphic head phantom

Lead‐free radiation‐shielding materials are used for medical workers and patients. Among them, we used TCR in this study.^18^, ^20^, ^21^, ^22^, ^23^, ^24^, ^25^, ^26^, ^27^ The elemental composition of the TCR (wt%) was H: 1.0%, C: 6.5%, O: 0.5%, W: 90.0%, and others: 2%. The TCR was 0.5 mm thickness. This TCR has been shown to significantly attenuate radiation with a shielding ability equivalent to that of a 0.25‐mm‐thickness lead barrier. We created a plastic helmet with the TCR affixed inside the forehead, the left side of the head, and the occiput (which we label “cap alone”: CA). This helmet was designed based on the protective cap described in previous studies.^14^, ^15^ Considering that physicians have high exposure on the left side of the head in IR,^16^, ^17^ we created protective equipment that covers the left cheek and ear. We combined the CA and the left lateral face shield and labeled this combination “flap” (Figure 3). The weights of the CA and the left lateral face shield were 223 g and 96 g, respectively. We measured the shielding performance of this protective equipment in three patterns: no protection, the CA, and the flap. The shielding efficiency was calculated from the following formula:

(1) $$\begin{array}{ccc} & & \mathrm{Shielding}\mathrm{efficiency}\;\%\\ & & =\frac{\mathrm{Dose}\;\mathrm{without}\;\mathrm{the}\;\mathrm{protective}\;\mathrm{cap}\;(\mathrm{flap})-\;\mathrm{Dose}\;\mathrm{with}\;\mathrm{the}\;\mathrm{protective}\;\mathrm{cap}\;(\mathrm{flap})}{\mathrm{Dose}\;\mathrm{without}\;\mathrm{the}\;\mathrm{protective}\;\mathrm{cap}\;(\mathrm{flap})}\\ & & \times 100 \end{array}$$

## 3 RESULTS

Tables 2 and 3 summarize the AKR and shielding efficiencies for the three locations in fluoroscopy and cine modes. In cine mode at LAO60CAU40, the AKRs on the surface of the left side of the head and the left and right temporal lobes were 94.3 μGy/15s, 48.8 μGy/15 s, and 8.3 μGy/15 s, respectively. Both temporal lobes were exposed, with the exposure of the left temporal lobe being larger. The shielding efficiencies on the surface of the left side of the head and the left and right temporal lobes with the CA were 92.6%, 5.1%, and 0.5%, respectively, while the corresponding shielding efficiencies with the flap were 92.5%, 86.1%, and 52.7%. The flap can reduce radiation exposure of the left and right temporal lobes more than the CA.

**TABLE 2:** AKR (μGy/min) and shielding efficiency (%) of fluoroscopy mode on the surface of the left side of the head, the left temporal lobe, and the right temporal lobe at each X‐ray tube angle

		**LAO60**		**LAO60CAU40**	
**Fluoroscopy**	**Tube angles**	**AKR (μGy/min)**	**Shielding efficiency (％)**	**AKR (μGy/min)**	**Shielding efficiency (％)**
**Surface of the left side of the head**	Non‐protect	14.6		11.5	
	CA	1.0	93.3	1.0	91.6
	Flap	0.8	94.3	1.1	90.7
**Left temporal lobe**	Non‐protect	9.6		6.3	
	CA	9.4	2.0	6.1	4.1
	Flap	1.7	82.3	0.9	85.4
**Right temporal lobe**	Non‐protect	1.4		1.2	
	CA	1.4	1.4	1.1	2.6
	Flap	1.1	20.4	0.1	93.0

**TABLE 3:** AKR (μGy/15s) and shielding efficiency (%) of cine mode on the surface of the left side of the head, the left temporal lobe, and the right temporal lobe at each X‐ray tube angle

		**LAO60**		**LAO60CAU40**	
**Cine**	**Tube angles**	**AKR (μGy/15s)**	**Shielding efficiency (％)**	**AKR (μGy/15s)**	**Shielding efficiency (％)**
**Surface of the left side of the head**	Non‐protect	78.2		94.3	
	CA	3.2	96.0	7.0	92.6
	Flap	3.0	96.2	7.1	92.5
**Left temporal lobe**	Non‐protect	45.5		48.8	
	CA	43.8	3.8	46.3	5.1
	Flap	7.2	84.3	6.8	86.1
**Right temporal lobe**	Non‐protect	5.9		8.3	
	CA	5.8	1.7	8.3	0.5
	Flap	4.7	20.6	3.9	52.7

## 4 DISCUSSION

In this study, we investigated the radiation exposure of the brain of a physician in IR by using phantom. The exposures of the left and right temporal lobes were measured at LAO. In cardiovascular procedures, we see many patients with high body mass index (BMI). These patients will increase the scattered radiation throughout the room. When the C‐arm is set at LAO60 and LAO60CAU40 for a large patient in cardiovascular procedures, the X‐ray system is automatically adjusted to a high tube voltage and current, which increases the scattered radiation and exposure of physicians. In addition, there is a possibility of increased exposure due to prolonged examination time such as for ablation and an intracardiac electrocardiogram.

We evaluated the shielding efficiencies of head protection devices. The cap alone was able to shield more than 90% at the surface of the left side of the head. However, the shielding efficiency of the cap alone was low at the left and right temporal lobes, less than 6%. These results indicate the cap alone can protect the surface of the head but cannot protect the brain. Meanwhile, the shielding efficiency of the flap on the left temporal lobe was more than 80%. Radiation exposure of the brain is quite affected by scatter radiation coming from the lower left side of the head. The flap can shield the lower left side of the head from scatter radiation and reduce exposure of the brain. The shielding efficiency of the left temporal lobe was 10% lower than the surface of the left side of the head. The reason of result may be caused by increasing scatter radiation from multiple directions.

Roguin et al. reported an increased risk of brain cancers; however, it is based on a small sample. In the present study, at LAO60CAU40 in cine mode, the left temporal lobe is exposed to 50 μGy per shot, meaning 100 shots can be as high as 5 mGy. Endo et al. reported that the dose for the left side of the left eye for cardiologists was 20.4 ± 9.2 mSv/year.^28^ Haga et al. reported this dose as 6.3 ± 5.1 mSv/6‐month.^29^ On the basis of these reports, we consider that the surface of the left side of the head is exposed to a similar dose. This study indicates that the exposure of the left temporal lobe might be 40% less compared to the surface of the left side of the head. Even so, the left temporal lobe is exposed to several mSv per year.

Ferrari P et al. examined radiation exposure of the physician's brain during IR through Monte Carlo simulation.^17^ The radiation exposure of the brain was determined throughout the simulation, with a particularly high exposure dose in the half of the brain nearer to the radiation source (front left down: 0.58 μGy/Gy·cm^2^ vs. front right down: 0.23 μGy/Gy·cm^2^). The present study showed similar results, with the left side of the brain having higher exposure than the right side. These results match the recently reported risk to the brain.^6^, ^7^, ^8^, ^9^ Therefore, brain protection may be necessary.

In the BRAIN study, protective surgical caps reduced the exposure of the surface of the left side of the head and the forehead to less than 50%.^14^ However, their study measured the exposure of the surface of the head, not the brain. Kirkwood ML et al. reported that a protective surgical cap reduced radiation exposure in the temporal position (on the surface of the head) by 71%, but only by 7.2% in the upper brain and 1.4% in the middle brain.^15^ In the present study, the cap alone reduced the dose on the surface of the head, but not the brain. In cine mode, the cap alone reduced exposure at LAO60CAU40 by 92.6% for the surface of the left side of the head, 5.1% for the left temporal lobe, and 0.5% for the right temporal lobe, as shown in Table 3. The results of the BRAIN study match those of the present study. Thus, a cap alone cannot protect the brain. On the other hand the flap can reduce radiation exposure of the brain more than the cap alone. In cine mode, the flap reduced exposure at LAO60CAU40 by 92.5% for the surface of the left side of the head, 86.1% for the left temporal lobe, and 52.7 % for the right temporal lobe, as shown in Table 3. Therefore, it is very important to determine the radiation exposure of the brain, and adding left‐cheek protection to the flap can significantly reduce this exposure.

Guni E et al. reported exposure of the brain and the shielding efficiency of a protective flap.^30^ The purpose of their study was to reduce exposure of the brain using a protective flap. The present study also shows that the reduction in exposure is superior in the left temporal lobe with a flap compared to without. However, the shielding efficiencies in the present study differ from their results, except for the left temporal lobe. In their study, they only measured PA, while we measured at LAO that is commonly used in clinical procedures. LAO is expected to change the angle of the scattered radiation to the brain of the physician and therefore increase the dose.

Physicians stand on the right side of a patient during IR, and the left side of the head is the closest to the primary X‐ray beam and scatter radiation. Physicians have long‐term exposure to low doses from many examinations for many years.^2^ The present study investigates that the brain is exposed to radiation, which may affect the physician. The flap can greatly reduce the exposure of the brain. We further suggest the flap cover the left cheek because a protective cap alone cannot reduce radiation exposure of the brain.

## 5 CONCLUSIONS

The present study clarified the exposure of the brain at LAO, which is a high exposure angle in IR. The head protection cap can protect the surface of the head, while it cannot reduce the exposure of the brain. It is possible to reduce the exposure of the brain by adding a flap.

## CONFLICT OF INTEREST

Hajime Monzen received a research donation from Hayakawa Rubber Co., Ltd., Tokyo, Japan.

## AUTHORS’ CONTRIBUTIONS

S Hattori, H Monzen, and H Kosaka were associated with concept and design. S Hattori, M Tamura, and H Kosaka took the measurements. S Hattori, H Kosaka, and Y Nakamura analyzed the data. S Hattori, H Monzen, M Tamura, H Kosaka, and Y Nishimura prepared the manuscript. All authors read and approved the final manuscript.

## Data Availability

The data that support the findings of this study are available from the corresponding author upon reasonable request.
